# Effectiveness of antihypertensive drugs for secondary prevention of ischaemic stroke: a nationwide historic cohort study

**DOI:** 10.1136/bmjopen-2025-107816

**Published:** 2025-12-15

**Authors:** Julia Perrier, Amelie Gabet, Valérie Olié, Antoine Pariente, Christophe Tzourio, Julien Bezin

**Affiliations:** 1 INSERM U1219, BPH, AHeaD team, University of Bordeaux, Bordeaux, Nouvelle-Aquitaine, France; 2 Sante publique France, Saint-Maurice, Île-de-France, France; 3 Medical Pharmacology Department, CHU de Bordeaux, Bordeaux, Nouvelle-Aquitaine, France; 4 INSERM U1219, BPH, University of Bordeaux, Bordeaux, Nouvelle-Aquitaine, France; 5 CHU de Bordeaux, Bordeaux, Nouvelle-Aquitaine, France

**Keywords:** Drug Therapy, Stroke, EPIDEMIOLOGY

## Abstract

**Abstract:**

**Objectives:**

To evaluate the impact of various antihypertensive drugs on secondary stroke prevention in a real-life setting.

**Design:**

Nationwide historic cohort study.

**Setting:**

French healthcare system data (SNDS).

**Participants:**

Adults hospitalised for ischaemic stroke between 2014 and 2015 were followed up until December 2021 and stratified based on the presence of atrial fibrillation (AF).

**Outcome measures:**

Risk of stroke recurrence was assessed using a time-dependent Cox cause-specific model accounting for changes in drug exposure. We also investigated the risk of major adverse cardiovascular events (MACE) or all-cause death. Models were adjusted on stroke characteristics, coprescriptions and co-morbidities, at inclusion and across follow-up.

**Results:**

Among 54 764 patients without AF (median age 71; 46% women) and 17 960 with AF (median age 79; 51% women), stroke recurrence occurred in 11% and 13%, respectively. In non-AF patients, reduced recurrence risk was associated only with use of calcium channel blockers (adjusted HR (aHR) 0.91, 95% CI 0.86 to 0.97), thiazide diuretics (aHR 0.90, 95% CI 0. 83 to 0.97), loop diuretics (aHR 0.86, 95% CI 0.77 to 0.95) and potassium-sparing agents (aHR 0.83, 95% CI 0.70 to 0.98). In AF patients, only potassium-sparing agents (aHR 0.82, 95% CI 0.69 to 0.99) were associated with reduced recurrence risk. All antihypertensive drugs, apart from loop diuretics, were associated with a reduced risk of MACE or all-cause death.

**Conclusions:**

In this large cohort, only diuretics and calcium channel blockers were associated with a reduced risk of recurrent stroke. Most antihypertensive drugs, however, may be more effective in overall cardiovascular prevention.

**STRENGTHS AND LIMITATIONS OF THIS STUDY**

- The database includes approximately 99% of the French population and provides exhaustive information on reimbursed medications and hospital discharge diagnoses.
- Stroke was identified using an algorithm validated in this database, with an overall positive predictive value of 96.3%.
- The diagnosis of atrial fibrillation may be identified long after stroke, which may have impacted our stratification.
- Although some residual confounding may persist due to the absence of biological and lifestyle data, such bias is likely to be limited, as the results of the sensitivity analyses were consistent with those of the main analysis.

## Introduction

 The latest European Stroke Organisation guidelines on ischaemic stroke highlight the importance of lowering blood pressure to reduce the risk of stroke recurrence. Treatment to lower blood pressure is strongly recommended, with a blood pressure target of less than 130/80 mm Hg.^1^ By expert consensus, initiation of dual antihypertensive therapy is suggested, except where there is a potential risk of hypotension, as in the elderly and frail, or in those with borderline hypertension. The recommendations of the European Society of Cardiology (ESC),^2^ based on those of the European Society of Hypertension (ESH),^3^ also recommend dual therapy for initial management. This therapy combines an ACE inhibitor (ACEi) or an angiotensin II receptor blocker (ARB) with a thiazide or thiazide-like diuretic and/or a calcium channel blocker (CCB). The ESH guidelines highlight the similar efficacy of ACEi and ARB in reducing adverse cardiovascular events and mortality. They also emphasise that the combination of these two drug classes is not recommended, as it offers no additional benefit while increasing the risk of renal complications. CCBs appear to be the least effective treatment for lowering blood pressure but have a greater impact on stroke reduction. Diuretics are effective at low doses in reducing adverse cardiovascular events. In the context of stroke prevention, beta-blockers (BB) are less effective than other antihypertensive drugs, although few clinical trials have directly compared combinations of different drug classes. According to the guidelines, ARB combined with thiazide or thiazide-like diuretic or ACEi combined with CCBs seems to be more effective in reducing cardiovascular risk. The 2021 recommendations from the American Heart Association (AHA) and the American Stroke Association (ASA) largely align with those of the ESC regarding antihypertensive treatments, as they also recommend using an ACEi or an ARB in combination with a thiazide or thiazide-like diuretic and/or a CCB, particularly in patients with a history of hypertension. However, in the absence of a history of hypertension, the AHA/ASA guidelines place greater emphasis on lowering blood pressure itself, regardless of the specific choice of antihypertensive treatment, which should be adapted to the patient’s other comorbidities.^4^

All these guidelines are based on clinical trials. However, few clinical trials specifically focused on antihypertensive drugs in the secondary prevention of ischaemic stroke, the most notable being the perindopril protection against recurrent stroke study (PROGRESS trial).^5^ This trial demonstrated the efficacy of the combination of perindopril (an ACEi) and indapamide (a thiazide or thiazide-like diuretic) after a stroke compared with perindopril alone and placebo. Furthermore, clinical trial populations are often highly selected and treated under optimal conditions, frequently excluding patients with certain comorbidities such as renal or hepatic impairment, as well as elderly individuals. However, older adults are among the primary targets for secondary stroke prevention treatments, as the average age of stroke occurrence is 72 years. The median age of patients included in clinical trials is about 7 years younger than that of patients in observational studies following a stroke.^6^ As a result, the applicability of clinical trial results to the general population remains uncertain. Finally, stroke survivors often have multiple comorbidities requiring several preventive treatments. Since drug classes are evaluated individually in clinical trials, the overall benefit of combining multiple treatments remains incompletely studied. The efficacy observed in clinical trials may therefore differ from what is actually achieved in real-world settings. The aim of this study was to evaluate the effectiveness of all the different antihypertensive drug classes in secondary ischaemic stroke prevention, in France, between 2014 and 2021.

## Methods

This study is reported in accordance with the reporting of studies conducted using observational routinely collected health data statement for pharmacoepidemiology (RECORD-PE) guideline of the Enhancing the QUAlity and Transparency Of Health Research network.^7^

### Database and study population

This historic nationwide cohort study was conducted using data from the French reimbursement healthcare system (SNDS) that includes information on 99% of the French population. Briefly, the main information available is demographic data, drug reimbursements, ambulatory care reimbursements and medical data mainly relating to in-hospital stay information. This database has been described in greater detail elsewhere.^8^ By agreement of the French Data Protection Authority (CNIL), neither ethics committee approval nor informed consent was required for this observational study based on anonymised French medico-administrative databases.

Patients aged 18 years old and over, hospitalised and discharged for a first ischaemic stroke between 1 January 2014 and 31 December 2015 were included. Incident ischaemic stroke was defined as a first hospitalisation for ischaemic stroke without identified previous history of stroke (haemorrhagic or ischaemic) since 2005. Hospitalisation for ischaemic stroke was identified using the diagnosis codes validated in the French hospitalisation database (International Classification of Diseases 10th Revision, ICD-10 diagnosis code I63, I63.6 excludes).^9^ The date of discharge from hospital was the date of inclusion in our study and the start of follow-up. In the case of successive short-term rehospitalisations (ie, within a maximum of 7 days), the date of inclusion was the last date of discharge identified. Due to the lack of information on the drugs supplied, patients who were discharged to nursing homes or had successive rehospitalisations beyond 4 months after their first stroke discharge were excluded.

Because atrial fibrillation and flutter (AF) are a major risk factor for stroke with specific prevention using anticoagulants, the study population was stratified into two groups defined according to AF history at inclusion. Patients with a history of diagnosed AF or with a history of anticoagulant use were classified in the AF group. Diagnosed AF was identified within 2 years before stroke, through hospitalisation diagnosis (ICD-10 code I48), and anticoagulant use (supply with Anatomical Therapeutic Chemical classification system (ATC) codes: vitamin K antagonists (B01AA), thrombin inhibitors (B01AE) and Xa inhibitors (B01AF)).

### Outcomes

The main outcome was stroke recurrence identified by hospitalisations for stroke (ICD-10 diagnosis code I60 to I64). To assess the effectiveness of the recommended drugs in a broader cardiovascular prevention perspective, we additionally considered the occurrence of major adverse cardiovascular events (MACE) or all-cause death in secondary analyses. MACE were identified by hospitalisations for stroke or acute coronary syndrome (ICD-10 diagnosis code I200, I21, I22, I24) or by revascularisation (medical procedure for percutaneous coronary intervention or for coronary artery bypass grafts; codes presented in online supplemental table S1).

### Definition and measurement of exposure

All antihypertensive drug classes were identified through drug supplies using ATC codes (online supplemental table S1). All antihypertensive drug prescriptions lead to reimbursement in France and are therefore fully identifiable in the database.

For each identified drug supply, the treatment period started at the date of supply and stopped after 30 days for monthly packaging or 91 days for trimestral packaging. For non-unitary pharmaceutical forms such as oral solutions, the treatment period was set at 30 days. A 5% grace period has been added to these treatment periods to consider any delay between supply and taking the drug.

Overlaps of treatment periods concerning the same drugs were cumulated. In the case of a switch (different therapeutic class and no successive overlaps in the following 91 days), overlaps were not considered; the switch drug stopped on the date of the new therapeutic class supply. In the case of association (different therapeutic class and successive overlaps in the following 91 days), treatment periods were not modified. At the end of a previously defined treatment period and in the absence of an additional supply, patients were considered unexposed to the treatment, until another treatment supply.

### Statistical analysis

Description of the population and the adjustment variables was provided. The effectiveness of the different antihypertensive drug classes was assessed using a time-dependent Cox cause-specific survival model, considering death as a competing risk and allowing exposure and comorbidities to change over time. All studied antihypertensive drug classes were compared with non-exposure and included in the same model. Patients were followed from hospital discharge date (start of follow-up, index date) to the date of the outcome, the date of admission into a nursing home, the date of death or the end of follow-up (31 December 2021). Follow-up of non-AF patients was also censored at the date of AF diagnosis or at the starting date of anticoagulant treatment, as defined previously.

The following variables were considered for adjustment: age, sex, other recommended treatments for secondary prevention (lipid-lowering drugs, antiplatelet agents and anticoagulants), hospitalisation with obesity, alcohol use disorder, tobacco dependence, diabetes, dementia, depression, ischaemic heart diseases, cardiac arrhythmia, peripheral artery disease, heart failure, chronic renal failure, history of hypertension (in-hospital diagnoses or antihypertensive drug treatment), history of lipid-lowering drugs and antiplatelet agents, number of different drugs used in the 6 months before stroke and French social deprivation index.^10^ Additionally, as the time to treatment is important in stroke emergencies, the travel time between place of residence and admission hospital was considered. Length of hospital stays for stroke management and the presence of a stroke unit were considered as proxies of stroke severity. Comorbidities and history of treatment were identified in the previous 2 years before stroke and across follow-up.

Three sensitivity analyses were conducted. First, to assess the impact of misidentification of diagnosed hypertension on the results, an analysis was performed considering only patients with a history of hypertension at the time of stroke. Second, to assess the impact of a potential healthy-user bias, an analysis was performed considering adjustment for exposure to vitamin D (ATC code: A11CC05) as a proxy for healthy-user. Lastly, as the COVID-19 epidemic occurred during patient follow-up, an analysis censoring follow-up to 29 February 2020 was performed to ensure that the epidemic had no impact on the results.

Analysis was performed using SAS software (SAS Institute, V.9.4, North Carolina, USA). Travel time between place of residence and hospital of care was calculated using the tool Metric-OSRM distancer (Insee, Metric-OSRM distancer, contributors to OpenStreetMap https://www.openstreetmap.org/copyright and the OSRM project).

### Patient and public involvement

None.

## Results

### Population characteristics

**Figure 1:**
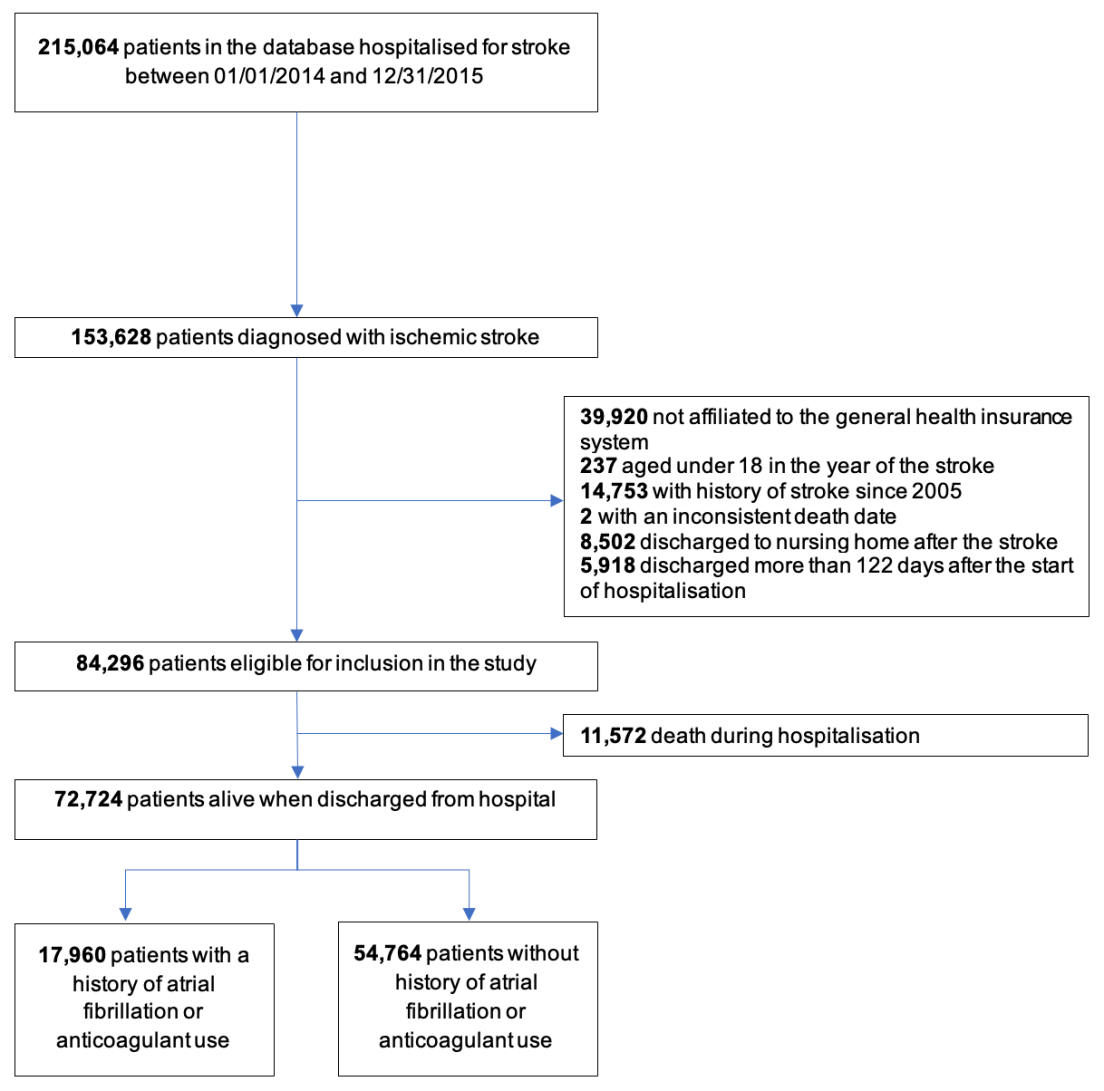
Flowchart of the study patient identification process.

Of the 153 628 patients hospitalised for ischaemic stroke between 2014 and 2015, 72 724 met the inclusion criteria including 54 764 non-AF patients and 17 960 AF patients (figure 1). Baseline characteristics of patients are detailed in table 1. Non-AF patients had a median age of 71 years and 46% were women. Over a median follow-up of 5.5 years, 5820 (11%) non-AF patients experienced stroke recurrence, and 15 937 (29%) MACE or all-cause death. AF patients appeared older with a median age of 79 years and 51% were women. Over a median follow-up of 4.6 years, 2355 (13%) AF patients experienced stroke recurrence and 8811 (49%) MACE or all-cause death. Cardiovascular comorbidities were fewer in non-AF than in AF patients (table 1).

**Table 1:** Baseline characteristics

Characteristics	Non-AF patients (N=54 764)		AF patients (N=17 960)	
	n*	(%)	n*	(%)
Median age (IQR)—year	71	(60–81)	79	(70–85)
Sex, women	25 138	(45.9)	9099	(50.7)
Comorbidities and history of drug use within 2 years before initial stroke				
Alcohol use disorder	2160	(3.9)	677	(3.8)
Anticoagulants	0	(0.0)	9097	(50.7)
Antiplatelet agents	18 329	(33.5)	7749	(43.2)
Atherosclerotic cardiovascular disease	3626	(6.6)	2871	(16.0)
Atrial fibrillation	0	(0.0)	6303	(35.1)
Cardiac arrhythmias	1853	(3.4)	2565	(14.3)
Chronic kidney disease	3258	(5.9)	2105	(11.7)
Dementia	1727	(3.2)	1061	(5.9)
Depression	8262	(15.1)	3085	(17.2)
Diabetes	11 253	(20.6)	4235	(23.6)
Heart failure	1519	(2.8)	2965	(16.5)
Hospitalisation with obesity	2804	(5.1)	1702	(9.5)
Hypertension	34 424	(62.9)	15 296	(85.2)
Lipid-lowering drugs	20 860	(38.1)	8561	(47.7)
Peripheral artery disease	3026	(5.5)	1782	(9.9)
Tobacco dependence	4853	(8.9)	2249	(12.5)
Hospital with a stroke unit	40 305	(73.6)	12 976	(72.3)
Transfer to rehabilitation and continuing care, during consecutive rehospitalisations	12 914	(23.6)	5536	(30.8)
Median number of different drugs used in the 6 months before initial stroke (IQR)	7	(3–12)	10	(6–15)
Travel time between place of residence and admission hospital				
(0–15) min	25 489	(46.5)	8472	(47.2)
(15–30) min	14 499	(26.5)	4885	(27.2)
(30–45) min	6289	(11.5)	2101	(11.7)
(45–60) min	2389	(4.4)	779	(4.3)
>60 min	2522	(4.6)	833	(4.6)
Not usable	3576	(6.5)	890	(5.0)
Median length of hospital stays (IQR)—days	9	(5–24)	13	(7–38)
French social deprivation index				
Missing	2928	(5.4)	464	(2.6)
1 (less deprived)	8873	(16.2)	3113	(17.3)
2	9854	(18.0)	3256	(18.1)
3	10 284	(18.8)	3534	(19.7)
4	10 829	(19.8)	3758	(20.9)
5 (more deprived)	11 996	(21.9)	3835	(21.4)

* Number of patients unless otherwise specified.

AF, atrial fibrillation.

### Dispensing after initial stroke

Among non-AF patients, 66% received antihypertensive drugs, compared with 79% of AF patients. The proportion of each antihypertensive drug class was similar regardless of AF status: ARB (16%), ACEi (30%), CCB (26%) and thiazide or thiazide-like diuretics (15%). However, AF patients were more likely to receive BB and loop diuretics than non-AF patients (46% vs 22% and 22% vs 6%, respectively).

### Drug effectiveness

**Figure 2:**
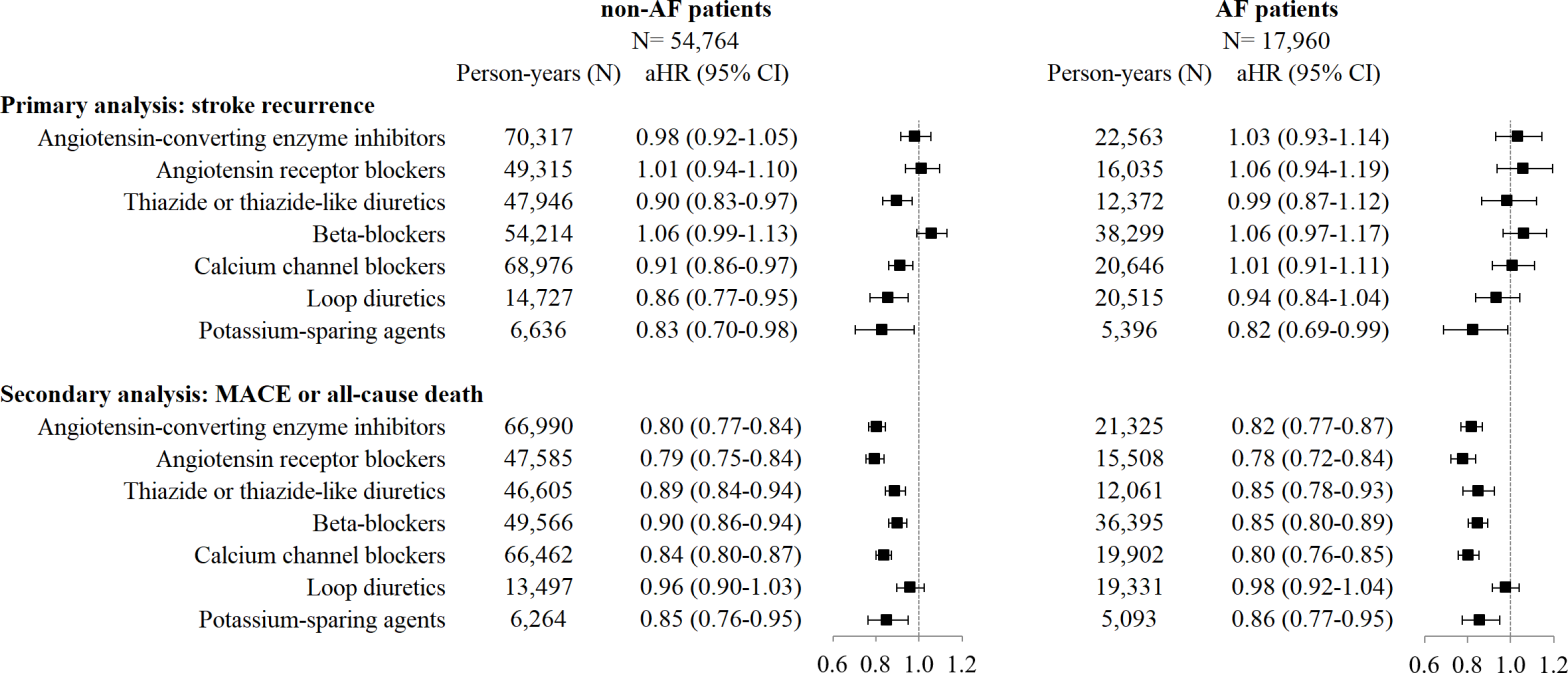
Effectiveness of antihypertensive drugs in AF and non-AF patients (time-dependent cause-specific Cox model) Estimates presented are aHRs with their 95% CIs. Models were adjusted on age, sex, social deprivation index, stroke characteristics, comorbidities and coprescriptions, at inclusion and across follow-up. AF, atrial fibrillation; aHR, adjusted HR; MACE, major adverse cardiovascular events.

No reduction in stroke recurrence was found associated with the use of ACEi, ARB or BB, neither in AF patients nor in non-AF ones. In non-AF patients, use of CCB (adjusted HR (aHR) 0.91; 95% CI 0.86 to 0.97), thiazide or thiazide-like diuretics (aHR 0.90; 95% CI 0.83 to 0.97), loop diuretics (aHR 0.86; 95% CI 0.77 to 0.95) and potassium-sparing agents (aHR 0.83; 95% CI 0.70 to 0.98) was associated with a decreased stroke recurrence. In AF patients, only use of potassium-sparing agents was associated with a decreased stroke recurrence (aHR 0.82; 95% CI 0.69 to 0.99) (figure 2, online supplemental table S2). In the secondary analyses considering broadly cardiovascular prevention, all antihypertensive drug classes except loop diuretics were found associated with a reduction of MACE or all-cause death, both in AF and non-AF patients (figure 2, online supplemental table S3).

### Sensitivity analyses

Sensitivity analyses performed in patients with a history of hypertension at the time of stroke (online supplemental table S4), adjusted on vitamin D use as a proxy for healthy-users (online supplemental table S5) and censoring the follow-up before the COVID period (online supplemental table S6), showed similar results.

## Discussion

In this large cohort of patients who had an ischaemic stroke followed at home for up to 8 years, only CCB and diuretics were found associated with a slightly reduced risk of stroke recurrence in non-AF patients. The only class of antihypertensive being associated with such decreased risk in AF patients was potassium-sparing diuretics. The results found when investigating broadly cardiovascular prevention were, however, much more reassuring. All antihypertensive drugs, except loop diuretics, were found associated with a reduced risk of MACE or all-cause death both in AF and non-AF patients. Taken together, the results we here present highlight that antihypertensive drugs could be more considered as important cardiovascular prevention drugs than as actual stroke prevention drugs.

Recent reviews and meta-analyses examined studies on blood pressure, which remains one of the major modifiable factors in stroke prevention. While lowering blood pressure too much can be harmful for patients in terms of rehabilitation,^11^ these reviews and meta-analyses highlighted the benefits of antihypertensive drugs after a stroke to reduce the risk of recurrence.^12^,^14^ It also reported that thiazide diuretics appeared to be the most effective antihypertensive drugs. This result was confirmed here in non-AF patients, as well as the effectiveness of CCB and other diuretics. However, in AF patients, we found that the use of antihypertensive drugs had no effect on the occurrence of recurrent stroke. Although statistically significant due to the nationwide nature of this study, these results remain of low clinical relevance for secondary stroke prevention. Regarding renin-angiotensin-system-acting agents also included in guidelines,^3 4^ no effectiveness of the ACEi or ARB was shown in this study specifically in terms of stroke recurrence. In the cohort of patients who had a stroke from the PROGRESS clinical trial^5^—a major trial on antihypertensive treatment after stroke— the combination of perindopril (ACEi) and indapamide (thiazide diuretic) was more effective than perindopril alone or placebo. While our cohort does not confirm the effectiveness of ACEi in reducing stroke recurrence, nor the lower efficacy of perindopril alone observed in the clinical trial, we found a slight decrease associated with the use of thiazide diuretics in poststroke treatment. The combination tested in the PROGRESS trial included a thiazide diuretic, but the effect of indapamide alone was not assessed. Overall, thiazide diuretics and CCBs may play a more significant role in secondary stroke prevention than ACEi or ARB. However, regarding MACE and all-cause mortality, our findings appeared reassuring for all antihypertensive drug classes (except for loop diuretics).

The database used represents almost 99% of the French population and contains all drug reimbursements and hospitalisation diagnoses. However, this database does not provide information on treatment during hospital or nursing home stays. This lack of information required us to exclude patients not discharged home after stroke. Thus, the results presented here may not generalise to this presumably older, more severely affected or higher-risk population. In addition, identifying AF in this database is challenging, as not all AF is treated with anticoagulants, especially patients with high bleeding risk or advanced renal disease, but also because AF may be identified late after stroke. Although this identification is a limitation of our study and could lead to misclassification of patients between groups, we nevertheless found a population comparable to that of the Dijon stroke registry, in France.^15^ Aside from the questionable identification of AF, a major advantage of this study is the identification of stroke using an algorithm validated in this database, with an overall positive predictive value of 96.3%.^9^ However, despite this performance in identifying strokes, the severity and aetiology cannot be directly identified. We approximated them using information on care pathways, but residual misclassification bias cannot be ruled out. Another limitation of this database is that it does not include clinical data such as blood pressure, cholesterol or data on lifestyle habits. This lack of data could have biased the identification of hypertension and explained the difference observed for antihypertensive drugs, which is why a sensitivity analysis was performed only in patients hospitalised for hypertension before the start of follow-up. The results of this sensitivity analysis were unchanged from those of the main analysis. The lack of blood pressure measurements also led to an inability to identify hypotension. Hypotension can be caused by suboptimal treatment in some patients, which we were unable to address due to the lack of clinical details. Furthermore, the blood pressure targets may also be set too low for some patients as older patients,^16^ which could explain the differences observed between antihypertensive drugs.^17^ In addition, the benefits found for other recommended drugs for secondary prevention of stroke (lipid-lowering drugs and antithrombotic drugs; online supplemental table S2 and S3) were in line with those of randomised clinical trials, ruling out the presence of major confounding bias in the analyses. The effects of antihypertensive drugs could also be masked by the greater efficacy of these other preventive treatments, and thus explain the lack of associations found in our results, especially for ACEi and ARB. Although it has already been shown that the efficacy of secondary prevention increases with the number of drugs taken, the contribution of ACEi or ARB in combination with lipid-lowering drugs, antiplatelet agents and CCB is negligible compared with the combination without ACEi or ARB, which is in line with our results.^18^ Furthermore, the concomitant use of multiple classes of antihypertensive drugs may produce attenuating effects, potentially contributing to the lack of association observed for stroke prevention. Conversely, the findings for MACE and all-cause mortality support our approach. Otherwise, patients compliant with secondary prevention may overall have healthier behaviours and lifestyle habits than those who do not comply, which would overestimate the effect of treatment on outcomes.^19^ To assess this potential bias, we conducted a sensitivity analysis by adjusting for vitamin D intake, which can also be considered as a proxy for healthy-user given its use in the prevention of fractures in the elderly,^20^,^22^ and the results were similar to those of the main analysis. Finally, the COVID period can impact dispensing and access to care during the follow-up, so we conducted a sensitivity analysis stopping the follow-up on 29 February 2020. The results of the latter were also in the same trend as previous analyses.

## Conclusion

These findings suggest that antihypertensive drugs may be more effective in overall cardiovascular prevention than in specifically preventing ischaemic stroke recurrence. While guidelines recommend combination of ACEi or ARB with a thiazide diuretic or CCB for blood pressure control after stroke, our results confirm the effectiveness for stroke recurrence only for thiazide diuretics and CCB in non-AF patients. Overall, our study underscores the pivotal role of CCB and diuretics in secondary stroke prevention but highlights the broader cardiovascular benefits of all antihypertensive drugs.

## Supplementary material

10.1136/bmjopen-2025-107816 Supplementary file 1

## Data Availability

Data may be obtained from a third party and are not publicly available.
