# Supplementary material for: Effectiveness of antihypertensive drugs for secondary prevention of ischaemic stroke: a nationwide historic cohort study
Source: BMJ Open. 2025 Dec 15;15(12):e107816. doi: 10.1136/bmjopen-2025-107816 (PMC12706251; doi:10.1136/bmjopen-2025-107816)
Supplement: Supplementary file 1 [file bmjopen-15-12-s001.docx]

**Supplementary material**

**Supplementary Tables**

Table S1. List of selected covariates and corresponding identification codes

Table S2. Primary analysis. Effectiveness of recommended drugs for secondary prevention on stroke recurrence, in AF and non-AF patients (time-dependent cause-specific cox model).

Table S3. Secondary analysis. Effectiveness of recommended drugs for secondary prevention on MACE or all-cause death, in AF and non-AF patients (time-dependent cause-specific cox model).

Table S4. Sensitivity analysis. Effectiveness of recommended drugs for secondary prevention on stroke recurrence, in AF and non-AF patients with history of hypertension (time-dependent cause-specific cox model).

Table S5. Sensitivity analysis. Effectiveness of recommended drugs for secondary prevention on stroke recurrence, in AF and non-AF patients, adjusted on vitamin D (time-dependent cause-specific cox model).

Table S6. Sensitivity analysis. Effectiveness of recommended drugs for secondary prevention on stroke recurrence, in AF and non-AF patients, censored before the COVID period (time-dependent cause-specific cox model).

Table S1. List of selected covariates and corresponding identification codes

|  | Sources | Codes |
| --- | --- | --- |
| Comorbidities: |  |  |
| Alcohol use disorder | Hospital discharge diagnosis (ICD-10) or registration for long-term disease (ICD-10) | F10, K70, T51, E24.4, F10, G31.2, G62.1, G72.1, I42.6, K29.2, K70, K86.0, T51, Z50.2, Z71.4, Z72.1, C70, C71, C79.3, C79.4, D32, D33, D42, D43, G04, G05, G06, G09, G12, G13, G24, G25, G26, G31, G32, G35, G36, G37, G46, G80, G81, G82, G83, G91, G93, G95 |
|  | Drug dispensing (ATC) | N07BB, M03BX01 |
|  | French biology code | 0519 |
|  | French medical procedure classification (CCAM) | 4101353, 401A011, 4164566, 401A012, 4184899, 401A013, 4169670, 401A014, 4107723, 401A021, 4118193, 401A022, 4122473, 401A022, 4179540, 401A04, 4111854, 401B01, 4147668, 401B02, 4130136, 401B03, 4122757, 401B04, 4174323, 4159619, 4195615, 401C01, 4142530, 401C02, 4183434, 401C03, 4168966, 401C04, 4113920, 4252810, 402A01, 4255116, 402A02, 4263950, 402A03, 4261051, 402A04, 4233570, 402C, 4325302, 401D011, 4371408, 401D012, 4342654, 401D013, 4327382, 401D014, 4329040, 401D015, 4324739, 401D016, 4300348, 401D017, 4321630, 4152847, 4375613, 401D020, 4375116, 401D021, 4371555, 401D022, 4359293, 401D023, 4308597, 401D031, 4339681, 401D032, 4309674, 401D041, 4307824, 401E011, 4302152, 401E012, 4389845, 401E011, 4348622, 401E012, 4307994, 401E021, 4326431, 402F011, 4371704, 402F012 |
| Anticoagulant | Drug dispensing (ATC) | B01AA, B01AE, B01AF |
| Atherosclerotic cardiovascular disease | Hospital discharge diagnosis (ICD-10) or registration for long-term disease (ICD-10) | I20, I21, I22, I23, I24, I25 |
|  | French medical procedure classification (CCAM) | DDAA002, DDAF001, DDAF003, DDAF004, DDAF006, DDAF007, DDAF008, DDAF009, DDAF010, DDFF001, DDFF002, DDPF002, DDMA002, DDMA003, DDMA004, DDMA005, DDMA006, DDMA007, DDMA008, DDMA009, DDMA011, DDMA012, DDMA013, DDMA015, DDMA016, DDMA017, DDMA018, DDMA019, DDMA020, DDMA021, DDMA022, DDMA023, DDMA024, DDMA025, DDMA026, DDMA027, DDMA028, DDMA029, DDMA030, DDMA031, DDMA032, DDMA033, DDMA034, DDMA035, DDMA036, DDMA037, DDMA038 |
| Atrial fibrillation | Hospital discharge diagnosis (ICD-10) or registration for long-term disease (ICD-10) | I48 |
| Cardiac failure | Hospital discharge diagnosis (ICD-10) or registration for long-term disease (ICD-10) | I50, I11.0, I13.0, I13.2, I13.9, K76.1 |
| Chronic kidney disease | Hospital discharge diagnosis (ICD-10) or registration for long-term disease (ICD-10) | N04, N07, N08, N11, N14, N15, N16, N18, N19, N25, N26, N27, N28, N29, Q61, Z94.0 |
|  | French medical procedure classification (CCAM) | JVJB001, JVJF004, JVJF008, JVRP004, JVRP007, JVRP008, YYYY007, JAEA003, HNEA002 |
|  | Hospital diagnosis related group | 11K021, 11K022, 11K023, 11K024, 11K02J, 28Z01Z, 28Z02Z, 28Z03Z, 28Z04Z, 28Z05Z, 28Z06Z, 27C06Z, 27C061, 27C062, 27C063, 27C064, 24M39Z, 11M171, 11M172, 11M173, 11M174 |
|  | Drug dispensing (ATC) | L04AA06, L04AA10, L04AA18, L04AD01, L04AD02, L04AX01 |
| Dementia | Hospital discharge diagnosis (ICD-10) or registration for long-term disease (ICD-10) | G30, F00, F01, F02, F03, F05.1  (excl. F023, F024) |
|  | Drug dispensing (ATC) | N06DA02, N06DA03, N06DA04, N06DX01 |
| Depression | Hospital discharge diagnosis (ICD-10) or registration for long-term disease (ICD-10) | F32, F33, F34, F38, F39 |
|  | Drug dispensing (ATC) | N06A |
| Diabetes | Hospital discharge diagnosis (ICD-10) or registration for long-term disease (ICD-10) | E10 E11 E12 E13 E14 G590 G632 G730 G990 H280 H360 I792 L97 M142 M146 N083 |
|  | Drug dispensing (ATC) | A10, excl. A10BX06 |
| Lipid-lowering drugs | Drug dispensing (ATC) | C10 |
| Dysrhythmias | Hospital discharge diagnosis (ICD-10) or registration for long-term disease (ICD-10) | I44, I45, I47, I49, R00 |
| Hospitalization for obesity | Hospital discharge diagnosis (ICD-10) or registration for long-term disease (ICD-10) | E65, E66 |
|  | French medical procedure classification (CCAM) | HFCC003, HFCA001, HFMC007, HFMA009, HFFC018, HFFA011, HFFC004, HFFA001, HGCC027, HGCA009, HMFA011, HFMC008 |
| Hypertension | Hospital discharge diagnosis (ICD-10) or registration for long-term disease (ICD-10) | I10, I11, I12, I13, I15, I16.74 |
|  | Drug dispensing (ATC) | C02, C03, C07, C08, C09 |
| Peripheral artery disease | Hospital discharge diagnosis (ICD-10) or registration for long-term disease (ICD-10) | I70, I71, I72, I73, I74 |
| Tobacco dependence | Hospital discharge diagnosis (ICD-10) or registration for long-term disease (ICD-10) | F17, J41, J42, J43, J44, T65.2, Z50.8, I73.1, Z58.7, Z71.6, Z72.0, J96.1 |
|  | Drug dispensing (ATC) | N07BA, N06AX12, R03BB05, R03BB04, R03BB07, R03BB06, R03AC18, R03AC19, R03AL03, R03AL04, R03AL05, R03AL06, R03AL08, R03AL09 |
| Events: |  |  |
| MACE | Hospital discharge diagnosis (ICD-10) | I20.0, I21, I22, I24, I60- I64 |
|  | French medical procedure classification (CCAM) | DDAA002, DDAF001, DDAF003, DDAF004, DDAF006, DDAF007, DDAF008, DDAF009, DDAF010, DDFF001, DDFF002, DDPF002, DDMA002, DDMA003, DDMA004, DDMA005, DDMA006, DDMA007, DDMA008, DDMA009, DDMA011, DDMA012, DDMA013, DDMA015, DDMA016, DDMA017, DDMA018, DDMA019, DDMA020, DDMA021, DDMA022, DDMA023, DDMA024, DDMA025, DDMA026, DDMA027, DDMA028, DDMA029, DDMA030, DDMA031, DDMA032, DDMA033, DDMA034, DDMA035, DDMA036, DDMA037, DDMA038 |
| Stroke | Hospital discharge diagnosis (ICD-10) | I60- I64 |
| Drugs of interest: |  |  |
| Angiotensin receptor blockers | Drug dispensing (ATC) | C09C, C09D |
| Angiotensin-converting enzyme inhibitors | Drug dispensing (ATC) | C09A, C09B |
| Antiplatelet agents | Drug dispensing (ATC) | B01AC, C10BX06, C10BX02 |
|  | Drug dispensing (French reimbursement code) | 3400930007167, 3400930007174, 3400930007181, 3400930078181, 3400930180549, 3400930192764, 3400931893639, 3400931893868, 3400932012077, 3400932144341, 3400932400935, 3400932452804, 3400932470891, 3400932517602, 3400932703555, 3400932703616, 3400932703784, 3400932901807, 3400932911042, 3400932926701, 3400932926879, 3400933438647, 3400933588540, 3400934558016, 3400934558184, 3400935091932, 3400935092014, 3400935092182, 3400935092243, 3400935125224, 3400936416048, 3400936416109, 3400936416277, 3400936987395, 3400937376983, 3400955002635, 3400955002642, 3400955002659, 3400955013662, 3400955013679, 3400955013686, 3400955466666, 3400955572855, 3400955794189, 3400956092666, 3400958265785 |
| Beta-blockers | Drug dispensing (ATC) | C07 |
| Calcium channel blockers | Drug dispensing (ATC) | C07FB02, C07FB03, C08, C09BB, C09DB, C10BX03 |
| Fibrates | Drug dispensing (ATC) | C10AB |
| Loop diuretics | Drug dispensing (ATC) | C03C, C03EB |
| Other lipid-lowering agents | Drug dispensing (ATC) | C10AX |
| Others antihypertensives drugs | Drug dispensing (ATC) | C02A, C02CA, C02DC01, C09X: discontinued in 2017 |
|  |  |  |
|  |  |  |
| Potassium-sparing agents | Drug dispensing (ATC) | C03D, C03E |
| Statins | Drug dispensing (ATC) | C10AA, C10BA, C10BX |
| Thiazide or thiazide-like diuretics | Drug dispensing (ATC) | C03A, C03B, C02LA01, C03EA, C07B, C07C, C07D, C08G, C09BA, C09DA, C09DX01, C09XA52 |
| Thrombin inhibitors | Drug dispensing (ATC) | B01AE |
| Vitamin K antagonists | Drug dispensing (ATC) | B01AA |
| Xa inhibitors | Drug dispensing (ATC) | B01AF |

Abbreviations: ATC: anatomical therapeutic chemical classification system; ICD-10: International Classiﬁcation of Diseases 10^th^ revision; MACE: Major Adverse Cardiac Events.

Table S2. Primary analysis. Effectiveness of recommended drugs for secondary prevention on stroke recurrence, in AF and non-AF patients (time-dependent cause-specific cox model).

|  | **Non-AF patients (n=54,764)** | |  | **AF patients (n=17,960)** | |
| --- | --- | --- | --- | --- | --- |
|  | **Person-year** | **aHR (95% CI)** |  | **Person-year** | **aHR (95% CI)** |
| Antihypertensive drugs |  |  |  |  |  |
| Angiotensin-converting enzyme inhibitors | 70,317 | 0.98 (0.92-1.05) |  | 22,563 | 1.03 (0.93-1.14) |
| Angiotensin receptor blockers | 49,315 | 1.01 (0.94-1.10) |  | 16,035 | 1.06 (0.94-1.19) |
| Thiazide or thiazide-like diuretics | 47,946 | 0.90 (0.83-0.97) |  | 12,372 | 0.99 (0.87-1.12) |
| Beta blockers | 54,214 | 1.06 (0.99-1.13) |  | 38,299 | 1.06 (0.97-1.17) |
| Calcium channel blockers | 68,976 | 0.91 (0.86-0.97) |  | 20,646 | 1.01 (0.91-1.11) |
| Loop diuretics | 14,727 | 0.86 (0.77-0.95) |  | 20,515 | 0.94 (0.84-1.04) |
| Potassium-sparing agents | 6,636 | 0.83 (0.70-0.98) |  | 5,396 | 0.82 (0.69-0.99) |
| Lipid-lowering drugs |  |  |  |  |  |
| Statins | 138,767 | 0.81 (0.76-0.87) |  | 40,584 | 0.76 (0.68-0.84) |
| Fibrates | 3,303 | 0.88 (0.70-1.10) |  | 1,071 | 0.73 (0.49-1.10) |
| Others | 4,298 | 0.75 (0.60-0.93) |  | 1,376 | 0.75 (0.53-1.07) |
| Antithrombotic drugs |  |  |  |  |  |
| Antiplatelet agents | 176,170 | 0.74 (0.69-0.80) |  | 18,106 | 0.80 (0.71-0.90) |
| Vitamin K antagonists |  | - |  | 27,181 | 0.51 (0.45-0.58) |
| Thrombin inhibitors |  | - |  | 3,673 | 0.48 (0.38-0.61) |
| Xa inhibitors |  | - |  | 25,113 | 0.47 (0.41-0.53) |

Abbreviations: AF: atrial fibrillation, aHR: adjusted hazard ratio, CI: confidence interval

Table S3. Secondary analysis. Effectiveness of recommended drugs for secondary prevention on MACE or all-cause death, in AF and non-AF patients (time-dependent cause-specific cox model).

|  | **Non-AF population (n=54,764)** | |  | **AF population (n=17,960)** | |
| --- | --- | --- | --- | --- | --- |
|  | **Person-year** | **aHR (95% CI)** |  | **Person-year** | **aHR (95% CI)** |
| Antihypertensive drugs |  |  |  |  |  |
| Angiotensin-converting enzyme inhibitors | 66,990 | 0.80 (0.77-0.84) |  | 21,325 | 0.82 (0.77-0.87) |
| angiotensin receptor blockers | 47,585 | 0.79 (0.75-0.84) |  | 15,508 | 0.78 (0.72-0.84) |
| Thiazide or thiazide-like diuretics | 46,605 | 0.89 (0.84-0.94) |  | 12,061 | 0.85 (0.78-0.93) |
| Beta blockers | 49,566 | 0.90 (0.86-0.94) |  | 36,395 | 0.85 (0.80-0.89) |
| Calcium channel blockers | 66,462 | 0.84 (0.80-0.87) |  | 19,902 | 0.80 (0.76-0.85) |
| Loop diuretics | 13,497 | 0.96 (0.90-1.03) |  | 19,331 | 0.98 (0.92-1.04) |
| Potassium-sparing agents | 6,264 | 0.85 (0.76-0.95) |  | 5,093 | 0.86 (0.77-0.95) |
| Lipid-lowering drugs |  |  |  |  |  |
| Statins | 133,029 | 0.65 (0.62-0.68) |  | 38,610 | 0.67 (0.63-0.71) |
| Fibrates | 3,226 | 0.64 (0.53-0.76) |  | 1,037 | 0.63 (0.49-0.81) |
| Others | 3,959 | 0.74 (0.63-0.85) |  | 1,221 | 0.91 (0.74-1.12) |
| Antithrombotic drugs |  |  |  |  |  |
| Antiplatelet agents | 169,832 | 0.47 (0.45-0.49) |  | 16,480 | 0.60 (0.56-0.64) |
| Vitamin K antagonists |  | - |  | 26,183 | 0.31 (0.29-0.33) |
| Thrombin inhibitors |  | - |  | 3,582 | 0.27 (0.24-0.32) |
| Xa inhibitors |  | - |  | 24,205 | 0.32 (0.30-0.35) |

Abbreviations: AF: atrial fibrillation, aHR: adjusted hazard ratio, CI: confidence interval, MACE: major adverse cardiovascular events.

Table S4. Sensitivity analysis. Effectiveness of recommended drugs for secondary prevention on stroke recurrence, in AF and non-AF patients with history of hypertension (time-dependent cause-specific cox model).

|  | **Non-AF patients (n=54,764)** | | **AF patients (n=17,960)** | |
| --- | --- | --- | --- | --- |
|  | **Primary analysis**  **aHR (95% CI)** | **Sensitivity analysis**  **aHR (95% CI)** | **Primary analysis**  **aHR (95% CI)** | **Sensitivity analysis**  **aHR (95% CI)** |
| Antihypertensive drugs |  |  |  |  |
| Angiotensin-converting enzyme inhibitors | 0.98 (0.92-1.05) | 0.97 (0.89-1.04) | 1.03 (0.93-1.14) | 1.00 (0.89-1.11) |
| Angiotensin receptor blockers | 1.01 (0.94-1.10) | 1.01 (0.93-1.10) | 1.06 (0.94-1.19) | 1.02 (0.90-1.16) |
| Thiazide or thiazide-like diuretics | 0.90 (0.83-0.97) | 0.90 (0.83-0.97) | 0.99 (0.87-1.12) | 0.97 (0.85-1.11) |
| Beta blockers | 1.06 (0.99-1.13) | 1.09 (1.02-1.16) | 1.06 (0.97-1.17) | 1.08 (0.98-1.19) |
| Calcium channel blockers | 0.91 (0.86-0.97) | 0.91 (0.85-0.97) | 1.01 (0.91-1.11) | 0.99 (0.90-1.10) |
| Loop diuretics | 0.86 (0.77-0.95) | 0.87 (0.78-0.97) | 0.94 (0.84-1.04) | 0.92 (0.82-1.03) |
| Potassium-sparing agents | 0.83 (0.70-0.98) | 0.83 (0.70-0.98) | 0.82 (0.69-0.99) | 0.78 (0.65-0.95) |
| Lipid-lowering drugs |  |  |  |  |
| Statins | 0.81 (0.76-0.87) | 0.82 (0.75-0.89) | 0.76 (0.68-0.84) | 0.76 (0.68-0.84) |
| Fibrates | 0.88 (0.70-1.10) | 0.88 (0.68-1.13) | 0.73 (0.49-1.10) | 0.75 (0.50-1.13) |
| Others | 0.75 (0.60-0.93) | 0.80 (0.64-0.99) | 0.75 (0.53-1.07) | 0.72 (0.50-1.04) |
| Antithrombotic drugs |  |  |  |  |
| Antiplatelet agents | 0.74 (0.69-0.80) | 0.70 (0.64-0.76) | 0.80 (0.71-0.90) | 0.79 (0.70-0.90) |
| Vitamin K antagonists | - | - | 0.51 (0.45-0.58) | 0.50 (0.44-0.57) |
| Thrombin inhibitors | - | - | 0.48 (0.38-0.61) | 0.46 (0.35-0.59) |
| Xa inhibitors | - | - | 0.47 (0.41-0.53) | 0.46 (0.40-0.53) |

Abbreviations: AF: atrial fibrillation, aHR: adjusted hazard ratio, CI: confidence interval

Table S5. Sensitivity analysis. Effectiveness of recommended drugs for secondary prevention on stroke recurrence, in AF and non-AF patients, adjusted on vitamin D (time-dependent cause-specific cox model).

|  | **Non-AF patients (n=54,764)** | | **AF patients (n=17,960)** | |
| --- | --- | --- | --- | --- |
|  | **Primary analysis**  **aHR (95% CI)** | **Sensitivity analysis**  **aHR (95% CI)** | **Primary analysis**  **aHR (95% CI)** | **Sensitivity analysis**  **aHR (95% CI)** |
| Antihypertensive drugs |  |  |  |  |
| Angiotensin-converting enzyme inhibitors | 0.98 (0.92-1.05) | 0.99 (0.92-1.06) | 1.03 (0.93-1.14) | 1.03 (0.93-1.15) |
| Angiotensin receptor blockers | 1.01 (0.94-1.10) | 1.02 (0.94-1.10) | 1.06 (0.94-1.19) | 1.07 (0.94-1.20) |
| Thiazide or thiazide-like diuretics | 0.90 (0.83-0.97) | 0.90 (0.83-0.97) | 0.99 (0.87-1.12) | 0.99 (0.86-1.12) |
| Beta blockers | 1.06 (0.99-1.13) | 1.06 (1.00-1.13) | 1.06 (0.97-1.17) | 1.07 (0.97-1.17) |
| Calcium channel blockers | 0.91 (0.86-0.97) | 0.92 (0.86-0.98) | 1.01 (0.91-1.11) | 1.01 (0.92-1.12) |
| Loop diuretics | 0.86 (0.77-0.95) | 0.86 (0.78-0.96) | 0.94 (0.84-1.04) | 0.94 (0.84-1.05) |
| Potassium-sparing agents | 0.83 (0.70-0.98) | 0.83 (0.70-0.98) | 0.82 (0.69-0.99) | 0.82 (0.69-0.99) |
| Lipid-lowering drugs |  |  |  |  |
| Statins | 0.81 (0.76-0.87) | 0.82 (0.76-0.88) | 0.76 (0.68-0.84) | 0.76 (0.69-0.85) |
| Fibrates | 0.88 (0.70-1.10) | 0.88 (0.70-1.11) | 0.73 (0.49-1.10) | 0.74 (0.50-1.11) |
| Others | 0.75 (0.60-0.93) | 0.75 (0.61-0.93) | 0.75 (0.53-1.07) | 0.76 (0.53-1.08) |
| Antithrombotic drugs |  |  |  |  |
| Antiplatelet agents | 0.74 (0.69-0.80) | 0.76 (0.70-0.82) | 0.80 (0.71-0.90) | 0.81 (0.72-0.91) |
| Vitamin K antagonists | - | - | 0.51 (0.45-0.58) | 0.52 (0.46-0.58) |
| Thrombin inhibitors | - | - | 0.48 (0.38-0.61) | 0.49 (0.39-0.62) |
| Xa inhibitors | - | - | 0.47 (0.41-0.53) | 0.47 (0.41-0.54) |

Abbreviations: AF: atrial fibrillation, aHR: adjusted hazard ratio, CI: confidence interval

Table S6. Sensitivity analysis. Effectiveness of recommended drugs for secondary prevention on stroke recurrence, in AF and non-AF patients, censored before the COVID period (time-dependent cause-specific cox model).

|  | **Non-AF patients (n=54,764)** | | **AF patients (n=17,960)** | |
| --- | --- | --- | --- | --- |
|  | **Primary analysis**  **aHR (95% CI)** | **Sensitivity analysis**  **aHR (95% CI)** | **Primary analysis**  **aHR (95% CI)** | **Sensitivity analysis**  **aHR (95% CI)** |
| Antihypertensive drugs |  |  |  |  |
| Angiotensin-converting enzyme inhibitors | 0.98 (0.92-1.05) | 0.99 (0.92-1.07) | 1.03 (0.93-1.14) | 1.06 (0.95-1.19) |
| Angiotensin receptor blockers | 1.01 (0.94-1.10) | 1.01 (0.93-1.10) | 1.06 (0.94-1.19) | 1.05 (0.93-1.20) |
| Thiazide or thiazide-like diuretics | 0.90 (0.83-0.97) | 0.91 (0.84-0.99) | 0.99 (0.87-1.12) | 0.95 (0.83-1.10) |
| Beta blockers | 1.06 (0.99-1.13) | 1.04 (0.97-1.12) | 1.06 (0.97-1.17) | 1.07 (0.97-1.18) |
| Calcium channel blockers | 0.91 (0.86-0.97) | 0.92 (0.86-0.98) | 1.01 (0.91-1.11) | 1.00 (0.91-1.11) |
| Loop diuretics | 0.86 (0.77-0.95) | 0.86 (0.77-0.96) | 0.94 (0.84-1.04) | 0.90 (0.81-1.01) |
| Potassium-sparing agents | 0.83 (0.70-0.98) | 0.83 (0.70-0.99) | 0.82 (0.69-0.99) | 0.80 (0.65-0.97) |
| Lipid-lowering drugs |  |  |  |  |
| Statins | 0.81 (0.76-0.87) | 0.82 (0.76-0.88) | 0.76 (0.68-0.84) | 0.77 (0.69-0.86) |
| Fibrates | 0.88 (0.70-1.10) | 0.88 (0.69-1.12) | 0.73 (0.49-1.10) | 0.75 (0.49-1.13) |
| Others | 0.75 (0.60-0.93) | 0.70 (0.55-0.89) | 0.75 (0.53-1.07) | 0.64 (0.43-0.97) |
| Antithrombotic drugs |  |  |  |  |
| Antiplatelet agents | 0.74 (0.69-0.80) | 0.71 (0.66-0.77) | 0.80 (0.71-0.90) | 0.76 (0.67-0.86) |
| Vitamin K antagonists | - | - | 0.51 (0.45-0.58) | 0.47 (0.42-0.54) |
| Thrombin inhibitors | - | - | 0.48 (0.38-0.61) | 0.45 (0.35-0.58) |
| Xa inhibitors | - | - | 0.47 (0.41-0.53) | 0.42 (0.36-0.49) |

Abbreviations: AF: atrial fibrillation, aHR: adjusted hazard ratio, CI: confidence interval
