# Supplementary material for: Effectiveness of antihypertensive drugs for secondary prevention of ischaemic stroke: a nationwide historic cohort study
Source: BMJ Open. 2025 Dec 15;15(12):e107816. doi: 10.1136/bmjopen-2025-107816 (PMC12706251; doi:10.1136/bmjopen-2025-107816)
Supplement: Supplementary file 2 [file bmjopen-2025-107816.reviewer_comments.pdf]

PEER REVIEW HISTORY

BMJ Open publishes all reviews undertaken for accepted manuscripts. Reviewers are asked to complete a checklist review form and are provided with free text boxes to elaborate on their assessment. These free text comments are reproduced below.

ARTICLE DETAILS

Title (Provisional)

Effectiveness of antihypertensive drugs for secondary prevention of ischemic stroke: a nationwide historic cohort study

Authors

Perrier, Julia; gabet, amelie; Olié, Valérie; Pariente, Antoine; Tzourio, Christophe; Bezin, Julien

---

VERSION 1 - REVIEW

**Reviewer**	**1**
**Name**	**Sluis, Wouter**
**Affiliation**	**Utrecht University**
**Date**	**14-Sep-2025**
**COI**	**None**

I congratulate the authors on this extensive piece of work. It is reassuring to find out that treatment with anti-hypertensive agents is good for MACE and all-cause mortality and that there are signs that treatment with CCB or diuretics is beneficial in ischaemic stroke prevention. I do however have some concerns that need to be addressed

First of all, in the introduction the authors comment on trials conducted in populations that 'exceed' the general population in stroke severity or younger populations than the general population. However, nearly 13.000 patients were excluded because they were admitted to a nursing home (suggesting a higher stroke severity) or had a 'long' admission (which could also be due to complications during admittance). The authors need to comment on this in the discussion section whether (and how) this could have potentially biased their results.

Second, I think the 'limitations' of ICD-code based research should be mentioned throughout the discussion. One of the major flaws, the timing of the AF diagnosis for instance has been addressed, but there are more flaws like misclassification and limited clinical details (we

know nothing about stroke severity, stroke subtype (different etiologies could warrant different antihypertensives). Please elaborate on this in your discussion.

Have you considered to perform an overall analysis without comparing non-AF with AF patients?

The adjusted hazard ratio's for most antihypertensive are relatively small with narrow confidence intervals close to 1. Could you elaborate in the discussion whether you think this is clinically relevant?

**Reviewer**	**2**
**Name**	**Kaźmierski, Radosław**
**Affiliation**	**University of Zielona Gora, Neurology**
**Date**	**17-Sep-2025**
**COI**	**None**

Comprehensive global studies conducted since the 1990s, known as the Global Burden of Disease (GBD) project, have shown that stroke is the second or third leading cause of death worldwide, depending on the year. Moreover, stroke remains the primary cause of severe disability after the age of 40.

In view of these facts, the topic addressed by the authors is both timely and of high clinical importance. Their focus on the treatment of arterial hypertension—one of the principal risk factors for stroke—is particularly relevant and aligns well with current medical needs.

The authors base their analysis on epidemiological data from the French health-care system, which encompasses a substantial patient population and therefore appears appropriate for the conclusions drawn at the population level.

However, studies of this type inevitably yield rather general results and do not permit more detailed analyses. For example, it is unclear how many individuals in the cohort had excessively low blood pressure as a consequence of suboptimal treatment in patients with pre-existing extracranial or intracranial arterial stenoses, which could in turn precipitate secondary stroke.

In addition, the stated blood pressure target of 130/80 mmHg may be too low for older individuals with the aforementioned vascular changes and impaired cerebral autoregulation. It would be worthwhile for the authors to discuss this issue in more depth (see, for example, Li J et al., Evaluation of Optimal Diastolic Blood Pressure Range Among Adults With Treated Systolic Blood Pressure Less Than 130 mm Hg, JAMA Network Open, 2021;4(2):e2037554; and Beckett NS et al., Treatment of Hypertension in Patients 80 Years of Age or Older, New England Journal of Medicine, 2008;358:1887-98, among others).

Aside from these points, the manuscript is well written, clear, and engaging, and in my opinion, is suitable for publication

---

VERSION 1 - AUTHOR RESPONSE

Reviewer #1: Dr. Wouter Sluis, Utrecht University

Comments to the Author

I congratulate the authors on this extensive piece of work. It is reassuring to find out that treatment with anti-hypertensive agents is good for MACE and all-cause mortality and that there are signs that treatment with CCB or diuretics is beneficial in ischaemic stroke prevention. I do however have some concerns that need to be addressed

R1C1: First of all, in the introduction the authors comment on trials conducted in populations that 'exceed' the general population in stroke severity or younger populations than the general population. However, nearly 13.000 patients were excluded because they were admitted to a nursing home (suggesting a higher stroke severity) or had a 'long' admission (which could also be due to complications during admittance). The authors need to comment on this in the discussion section whether (and how) this could have potentially biased their results.

Authors: We thank the reviewer for this comment. We excluded these patients due to a lack of data on drug supply during stays in these establishments. We agree that this excluded population is likely to differ from the one studied in terms of severity or complications. However, successive readmissions within 7 days of discharge and those up to 122 days were considered, as these could be due to complications or the severity from the initial stroke. We have added this limit in the discussion section:

"In this large cohort of ischemic stroke patients followed at home for up to 8 years, only CCB and diuretics were found associated with a slightly reduced risk of stroke recurrence in non-AF patients."

"The database used represents almost 90% of the French population and contains all drug reimbursements and hospitalization diagnoses. However, this database does not provide information on treatment during hospital or nursing home stays. This lack of information required us to exclude patients not discharged home after stroke. Thus, the results presented here may not generalize to this presumably older, more severely affected, or higher-risk population "

R1C2: Second, I think the 'limitations' of ICD-code based research should be mentioned throughout the discussion. One of the major flaws, the timing of the AF diagnosis for instance has been addressed, but there are more flaws like misclassification and limited clinical details (we know nothing about stroke severity, stroke subtype (different etiologies could warrant different antihypertensives). Please elaborate on this in your discussion.

Authors: Thank you for that insightful comment. We fully agree that coding using ICD-10 could leads to misclassification, especially since we do not have the clinical details during hospital stays. This may be true for atrial fibrillation, but less for stroke, for which coding was evaluated in a French study with a PPV of 96.3% for ischaemic strokes. Although we have approximated the stroke severity using management in stroke unit and length of stay, residual biases cannot be ruled out. We have expanded this limit in the discussion section:

"In addition, identifying AF from this database is challenging, as not all AF is treated with anticoagulants, especially patients with high bleeding risk, or advanced renal disease, but also because AF may be identified late after stroke. Although this identification is a limitation of our study and could lead to misclassification of patients between the two groups, we nevertheless found a population that is comparable to that of the Dijon stroke registry, in France [15]. Aside from the questionable identification of AF, a major advantage of this study is the identification of stroke using an algorithm validated on this database, with an overall positive predictive value (PPV) of 96.3% [9]. However, despite this performance in identifying strokes, the severity and etiology cannot be directly identified. We approximated them using information on care pathway but residual misclassification bias cannot be ruled out. Another limitation of this database is that it does not include clinical data such as blood pressure, cholesterol, or data on lifestyle habits. […]"

R1C3: Have you considered to perform an overall analysis without comparing non-AF with AF patients?

Authors: This is an important point raised by the reviewer. When designing this project, we choose to stratify the studied population according to AF status. Discussions with the clinical experts included in the study highlighted the differences in drugs use according to guideline concerning antithrombotic agents. In order to comply with practices and guidelines, we considered it important to clearly distinguish this pathology. We therefore only performed analyses stratified by AF status.

R1C4: The adjusted hazard ratio's for most antihypertensive are relatively small with narrow confidence intervals close to 1. Could you elaborate in the discussion whether you think this is clinically relevant?

Authors: Thank you for that fair comment. The estimated values on stroke recurrence with antihypertensives are close to 1, which indicates that these treatments have probably few clinical relevance independently of other preventive treatments (lipid-lowering drugs, antithrombotic drugs). The statistical significance found is due to the high statistical power of the models on this nationwide population. We discussed this point as follows:

"In this large cohort of ischemic stroke patients followed at home for up to 8 years, only CCB and diuretics were found associated with a slightly reduced risk of stroke recurrence in non-AF patients."

"This result was confirmed here in non-AF patients, as well as effectiveness of CCB and other diuretics. However, in AF patients, we found that the use of antihypertensive drugs had no effect on the occurrence of recurrent stroke. Although statistically significant due to the nationwide nature of this study, these results remain of low clinical relevance for secondary stroke prevention."

"While our cohort does not confirm the effectiveness of ACEi in reducing stroke recurrence, nor the lower efficacy of perindopril alone observed in the clinical trial, we found a slight decrease associated with the use of thiazide diuretics in post-stroke treatment."

Reviewer #2: Prof. Radosław Kaźmierski, University of Zielona Gora, Poznan University of Medical Sciences

Comments to the Author

Comprehensive global studies conducted since the 1990s, known as the Global Burden of Disease (GBD) project, have shown that stroke is the second or third leading cause of death worldwide, depending on the year. Moreover, stroke remains the primary cause of severe disability after the age of 40.

In view of these facts, the topic addressed by the authors is both timely and of high clinical importance. Their focus on the treatment of arterial hypertension—one of the principal risk factors for stroke—is particularly relevant and aligns well with current medical needs.

The authors base their analysis on epidemiological data from the French health-care system, which encompasses a substantial patient population and therefore appears appropriate for the conclusions drawn at the population level.

However, studies of this type inevitably yield rather general results and do not permit more detailed analyses. For example, it is unclear how many individuals in the cohort had excessively low blood pressure as a consequence of suboptimal treatment in patients with pre-existing extracranial or intracranial arterial stenoses, which could in turn precipitate secondary stroke.

In addition, the stated blood pressure target of 130/80 mmHg may be too low for older individuals with the aforementioned vascular changes and impaired cerebral autoregulation. It would be worthwhile for the authors to discuss this issue in more depth (see, for example, Li J et al., Evaluation of Optimal Diastolic Blood Pressure Range Among Adults With Treated Systolic Blood Pressure Less Than 130 mm Hg, JAMA Network Open, 2021;4(2):e2037554; and Beckett NS et al., Treatment of Hypertension in Patients 80 Years of Age or Older, New England Journal of Medicine, 2008;358:1887-98, among others).

Aside from these points, the manuscript is well written, clear, and engaging, and in my opinion, is suitable for publication

Authors: Thank you for your insightful comment. It is true that our pharmaco-epidemiological study provides rather general results. The lack of clinical data, particularly on blood pressure measurements and pre-existing arterial stenoses, prevents us from identifying hypotension or fully assessing its potential contribution to the occurrence of our outcome of interest. Accounting for these factors would require a very specific database, which could reduce statistical power, while the clinical relevance would still be substantial.

We also acknowledge that the blood pressure target of 130/80 mmHg may be too low for older patients with vascular changes and impaired cerebral autoregulation, as highlighted in previous studies. This concern is frequently raised by clinicians, but cannot be addressed within the framework of our study. Additional studies including detailed blood pressure measurements and vascular assessments will be necessary to confirm and refine these findings.

We have added this hypothesis in the discussion:

"The lack of blood pressure measurements led also to an inability to identify hypotension. Hypotension can be caused by suboptimal treatment in some patients, which we were unable to address due to the lack of clinical details. Furthermore, the blood pressure targets may be also set too low for some patients as older patients [16], which could explain the differences observed between antihypertensive drugs [17]."

Additional Comments

Authors: We have made a mistake in the numbers presented in Table S4 in the Supplement for the sensitivity analysis in patients with history of hypertension. However, the error had very little effect on the numbers presented and therefore should have very little impact on the

understanding of the paper by reviewers. Please accept our apologies for this. We have corrected this error, and double-checked the other data presented and found no further mistakes.

VERSION 2 - REVIEW

**Reviewer**	**1**
**Name**	**Sluis, Wouter**
**Affiliation**	**Utrecht University**
**Date**	**14-Oct-2025**
**COI**	

I am satisfied with the comments by the authors and believe it has strengthened the discussion section, i have no further comments.

**Reviewer**	**2**
**Name**	**Kaźmierski, Radosław**
**Affiliation**	**University of Zielona Gora, Neurology**
**Date**	**11-Oct-2025**
**COI**	

The authors answered all my questions; no further comments.

**Reviewer**	**3**
**Name**	**Moinuddin, Mohammed**
**Affiliation**	**University of Lancashire, School of Medicine and Dentistry**
**Date**	**15-Nov-2025**
**COI**	**None**

This study aimed to evaluate the effectiveness of different antihypertensive drug classes for secondary ischemic stroke prevention in France. An interesting and valuable piece of research. Sound methodologies were adopted and statistical analysis was performed on the right track. I have made a few observations, as listed below:

Comment 1: In page 10, lines 24-26, the authors sated, “All studied antihypertensives drug classes were compared to non-exposure and included in the same model.” The main exposures and covariates were included in the model simultaneously. This approach may

introduce multicollinearity and exposure overlap because patients often receive multiple antihypertensive classes simultaneously. Therefore, the estimated hazard ratios may not accurately represent class-specific associations. It would be necessary to check for multicollinearity and overlapping treatments or mutually exclusive exposure effects.

Comment 2: The authors have frequently used the term 'effectiveness" in the article. I wonder if it is more suitable to use 'associated" due to the observational nature of the study.

VERSION 2 - AUTHOR RESPONSE

Reviewer #1: Dr. Wouter Sluis, Utrecht University

Comments to the Author

I am satisfied with the comments by the authors and believe it has strengthened the discussion section, I have no further comments.

Reviewer #2: Prof. Radosław Kaźmierski, University of Zielona Gora, Poznan University of Medical Sciences

Comments to the Author

The authors answered all my questions; no further comments.

Reviewer #3 (statistical reviewer): Dr. Mohammed Moinuddin, University of Lancashire

Comments to the Author

This study aimed to evaluate the effectiveness of different antihypertensive drug classes for secondary ischemic stroke prevention in France. An interesting and valuable piece of research. Sound methodologies were adopted and statistical analysis was performed on the right track. I have made a few observations, as listed below:

R3C1: In page 10, lines 24-26, the authors sated, "All studied antihypertensives drug classes were compared to non-exposure and included in the same model." The main exposures and covariates were included in the model simultaneously. This approach may introduce multicollinearity and exposure overlap because patients often receive multiple antihypertensive classes simultaneously. Therefore, the estimated hazard ratios may not accurately represent class-specific associations. It would be necessary to check for multicollinearity and overlapping treatments or mutually exclusive exposure effects.

Authors: Thank Dr. Mohammed Moinuddin for this comment, which is indeed very relevant. When designing the study, we also considered modelling the different combinations of antihypertensive drug classes separately in order to avoid the risks of multicollinearity and exposure overlap that you mention. However, exploration of treatment trajectories in our population revealed considerable heterogeneity: more than 350 possibilities (alone or in combination) from the first post-stroke supplying, and up to 700 possibilities over the entire follow-up period. Given this complexity, and the small sample sizes for many of these combinations, modelling mutually exclusive categories was very difficult to achieve. We therefore opted for a model with time-dependent exposures for each antihypertensive class, acknowledging the inherent limitations of mutual adjustment. We also considered the potential bias related to adjustment for other therapeutic classes (lipid-lowering drugs and antithrombotics). However, the associations

observed for these classes regarding cardiovascular prevention were consistent with results reported in clinical trials, which supports the plausibility of our modelling strategy.

We have clarified these methodological considerations in the revised manuscript and specified the associated limitations:

"The lack of blood pressure measurements led also to an inability to identify hypotension. Hypotension can be caused by suboptimal treatment in some patients, which we were unable to address due to the lack of clinical details. Furthermore, the blood pressure targets may be also set too low for some patients as older patients [16], which could explain the differences observed between antihypertensive drugs [17]. In addition, benefits found for other recommended drugs for secondary prevention of stroke (lipid-lowering drugs and antithrombotic drugs; Table S2 and Table S3 in the Supplement) were in line with that of randomized clinical trials, ruling out the presence of major confounding bias in the analyses. The effects of antihypertensives could also be masked by the greater efficacy of these other preventive treatments, and thus explain the lack of associations found in our results, especially for ACEi and ARB. Although it has already been shown that the efficacy of secondary prevention increases with the number of drugs taken, the contribution of ACEi or ARB in combination with lipid-lowering drugs, antiplatelet agents and CCB is negligible compared to the combination without ACEi or ARB, which is in line with our results [18]. **Furthermore, the concomitant use of multiple classes of antihypertensive drugs may produce attenuating effects, potentially contributing to the lack of association observed for stroke prevention. Conversely, the findings for MACE and all-cause mortality support our approach.** Otherwise, patients compliant with secondary prevention may overall have healthier behaviours and lifestyle habits than those who do not comply, which would overestimate the effect of treatment on outcomes [19]. To assess this potential bias, we conducted a sensitivity analysis by adjusting for vitamin D intake, which can also be considered as a proxy for healthy user given its use in the prevention of fractures in the elderly [20–22], and the results were similar to those of the main analysis."

R3C2: The authors have frequently used the term 'effectiveness" in the article. I wonder if it is more suitable to use 'associated" due to the observational nature of the study.

Authors: We appreciate your comment. From our point of view, effectiveness refers to real-life efficacy, which is the case here, even if the term association is probably more accurate. We have chosen to use the term 'effectiveness' in particular for the title, abstract and conclusion because we felt it was appropriate to summarize these observational results. However, we have used the term 'associated' as much as possible in the manuscript (particularly in the results section).

VERSION 3 - REVIEW

**Reviewer**	**3**
**Name**	**Moinuddin, Mohammed**
**Affiliation**	**University of Lancashire, School of Medicine and Dentistry**
**Date**	**29-Nov-2025**
**COI**	

I thank the authors for clarifying these concerns. I am satisfied with the current version of the manuscript. Congratulations!
